# Towards the high-resolution protein structure prediction. Fast refinement of reduced models with all-atom force field

**DOI:** 10.1186/1472-6807-7-43

**Published:** 2007-06-29

**Authors:** Sebastian Kmiecik, Dominik Gront, Andrzej Kolinski

**Affiliations:** 1 University of Warsaw, Faculty of Chemistry, Pasteura 1, 02-093 Warsaw, Poland

## Abstract

**Background:**

Although experimental methods for determining protein structure are providing high resolution structures, they cannot keep the pace at which amino acid sequences are resolved on the scale of entire genomes. For a considerable fraction of proteins whose structures will not be determined experimentally, computational methods can provide valuable information. The value of structural models in biological research depends critically on their quality. Development of high-accuracy computational methods that reliably generate near-experimental quality structural models is an important, unsolved problem in the protein structure modeling.

**Results:**

Large sets of structural decoys have been generated using reduced conformational space protein modeling tool CABS. Subsequently, the reduced models were subject to all-atom reconstruction. Then, the resulting detailed models were energy-minimized using state-of-the-art all-atom force field, assuming fixed positions of the alpha carbons. It has been shown that a very short minimization leads to the proper ranking of the quality of the models (distance from the native structure), when the all-atom energy is used as the ranking criterion. Additionally, we performed test on medium and low accuracy decoys built via classical methods of comparative modeling. The test placed our model evaluation procedure among the state-of-the-art protein model assessment methods.

**Conclusion:**

These test computations show that a large scale high resolution protein structure prediction is possible, not only for small but also for large protein domains, and that it should be based on a hierarchical approach to the modeling protocol. We employed Molecular Mechanics with fixed alpha carbons to rank-order the all-atom models built on the scaffolds of the reduced models. Our tests show that a physic-based approach, usually considered computationally too demanding for large-scale applications, can be effectively used in such studies.

## Background

Reliable high-resolution prediction of protein structure remains a formidable challenge and it becomes more and more evident that we are entering the era in which high-resolution predictions and molecular designs will make increasingly important contributions to biology and medicine [1,2]. The high-resolution models could be built by means of various comparative modeling procedures, although it is also sometimes possible to obtain good models in a template-free modeling of small globular proteins [2-5].

Determining and properly quantifying the properties that are characteristic for protein native structures are of primary importance for the construction of an accurate tool for the model quality assessment. Several different approaches to the optimal model selection have been proposed – such as the use of empirical or knowledge-based potentials [6,7] derived from the databases of experimental structures. More straightforward, although more expensive computationally, is the evaluation of conformational energy by means of Molecular Mechanics force fields [8-10]. Another approach to the model selection is the structural clustering, especially useful when large set of models must be assessed [11]. Finally, learning-based scoring functions can be developed using machine learning methods e.g. support vector machines [12], neural networks [13,14], etc.

It is widely believed that the native conformation of a protein corresponds to the global minimum of the free energy surface defined by the protein's conformational space and the molecular interactions. A straightforward protein modeling by the all-atom energy minimization remains impractical due to the high complexity of the interactions and astronomical size of the conformational space to be searched. Thus, most approaches used for exploring the protein's energy surface have resorted to essential simplifications in the description of the polypeptide chain geometry and definition of molecular interactions. Properly designed reduced models make possible very effective search of the protein's conformational space. Model simplifications, while beneficial in filtering out the majority of unrealistic structures, limit the degree of accuracy that can be achieved. In most contemporary approaches to protein structure prediction large sets of alternative models are built. Proper selection of the best model is in many cases as difficult as obtaining very good models (usually mixed with not so good models).

Even in the simplest case of protein structure prediction – comparative modeling, the exact structure of a target protein differs from its nearest structural template used in modeling. Such deviations can not be corrected on the low-resolution modeling level, a more detailed representation of the protein and more realistic force field are needed. Unfortunately, more complex energy functions produce more rough energy landscapes, which consequently makes sampling much more difficult. Thus, it seems reasonable to split the modeling process into two stages: fold assembly (in a simplified representation) followed by the model refinement/selection procedure, using a more detailed representation (preferentially all-atom) and a more exact interaction scheme.

The first attempts at using the all-atom modeling as a final stage of hierarchical approach were applied to GCN4 leucine zipper – a very simple homodimer coiled-coil consisting of two 33-mer monomers [3,4]. The simulations were held in the times when even short macromolecular simulations were hardly possible due to limitations of computer power. ~1 Å backbone RMSD (coordinate Root-Mean-Square Deviation from the native structure after the best superimposition) was achieved by means of reduced modeling of GCN4 leucine zipper, followed by a molecular dynamics annealing protocol [4]. Such improvement was possible only with the help of α-helical constraints applied to each residue. A decade after the pioneering work by Vieth et al. [4] molecular dynamics was still too expensive for significant protein structure refinements. More recently, explicit solvent molecular dynamics and implicit solvent energy calculations on 12 small, single-domain proteins allowed a successful ranking of the near-native conformations and the best structure selection from predictions generated by Rosetta method [8]. However, the simulations were unable to refine the best structures. *De novo *models produced by Rosetta were also subjected to molecular dynamics simulations performed in explicit water [15]. RMSD values of the starting models increased during the short simulations, but longer simulations appeared to generate tighter packing of helices and regularization of β-strands in some cases. Very encouraging result was also obtained by Simmerling et al. [5], who managed to significantly improve assembled on a lattice, low resolution structure of 29-mer CMTI-1 protein (3.7 Å from native). The final model had the correct packing of β-strands and was much closer to the native structure (2.2 Å).

Very interesting hierarchical approach to protein folding was developed by Levitt group [16]. First, a large set of compact decoys was generated on a very coarse-grained lattice. Then, fragments extracted from known structures were fitted to the lattice scaffolds. Subsequently an elaborated procedure for the model selection and evaluation was performed. Quite good structures were finally predicted. To some extent the present approach follows this idea, although the higher resolution lattice decoys enable a higher resolution modeling by the entire hierarchical scheme.

Currently, probably the most successful refinement procedures use the all atom force-field that focuses on the short range interactions and Monte Carlo minimization. Unfortunately, the methods consume a lot of computer power and can be used only for small protein domains [2]. The authors suggest that the primary bottleneck in a consistent high resolution prediction appears to be the conformational sampling. Insufficient sampling misses the native basin and a false minimum could be selected.

Here we show that by using a combination of a relatively high resolution sampling in a reduced conformational space, with the model selection by an all-atom detailed potentials and a high performance computing, the high resolution structure prediction can be achieved (less than 1.0 Å from native). To get such result the reduced models need to be diverse enough to cover the near-native subset of the conformational space. CABS (CA-CB-Side chain) modeling tool was employed for this purpose [17,18]. CABS model was successfully used by Kolinski-Bujnicki group during the CASP6 (Critical Assessment of Protein Structure Prediction) experiment – the average score of the models submitted by this group was the second best among about 200 groups participating. Interestingly, inspections of the simulation trajectories after publication of the target structures have shown, that there were always better models (frequently much better) than those submitted to the CASP6 server. The lack of specificity of the CABS force field in a 3 Å vicinity of the native structure, was the main reason of the poor model selection. In this range the CABS energy is poorly correlated with RMSD for the majority of proteins. During the CASP6 experiment the group mentioned above, did not have sufficient computer resources for the all-atom refinement. Also, the role of even brief all-atom refinement in the proper model selection was underestimated at that time by the authors. Nevertheless, several submitted models (comparative modeling using CABS) were of very high accuracy, similar to the accuracy of crystallographic structures (detailed results are available at CASP6 website [19]).

In the present work we have demonstrated that a short, all-atom minimization with fixed Cα positions can properly rank-order large sets of near native decoys generated by CABS. In this context it becomes apparent that critical for the high-resolution protein structure prediction is ability to generate sets of models that contain some near-native structures. In comparative modeling with CABS, it could be achieved by using restraints extracted from various templates with alternative alignments in the uncertain regions. To our knowledge, that's the first approach enabling a meaningful refinement of large protein domains. The procedure proposed here may also work for small proteins in the template-free modeling. In such cases very large and diverse sets of decoys need to be generated and properly clustered before the all-atom based model selection. To further evaluate the proposed method for model assessment and ranking, we also performed tests on models generated by MODELLER [20] – probably the most popular, versatile and quite accurate computational tool for comparative modeling. Such models are collected in the MOULDER testing set [12] – a comprehensive and well evaluated, present-day decoy set. Numerous state-of-the-art methods for model selection were tested using this set. Our method performed similarly well, or even better than majority of the other methods. Very rigorous criteria of the model ranking assessments were used to make this comparison.

## Results and discussion

### Construction of the CABS decoy sets

We tested the proposed model ranking protocol on large sets of near-native decoys. We constructed a benchmark of 7 proteins [21], which are representative in respect to their length and secondary structure content (Table 1). None of these proteins was present, or had detectable homologs, in the library of the protein fragments used for the backbone reconstruction (the test structures were added very recently to the PDB).

**Table 1:** The CABS decoys set

PDB ID	% α	% β	L	% of low energy structures
2gr8A	16	50	78	99
2cklA	44	14	98	93
2gmkA	19	41	103	75
2gu3A	19	48	128	79
2grrB	64	0	157	92
2cl4X	52	2	250	68
2cjpA	45	16	320	38

Particular columns contain: the PDB code, the fraction of alpha helices, the fraction of beta strands, the protein length and the fraction of correctly built structures (where the minimization did not result in abnormal high energy values).

Several studies have utilized different energy functions to discriminate the native structure among sets of decoys built in different ways [9,22-25]. Typically, the decoys have been generated by means of various threading procedures. Unfortunately, the decoys' sets built by threading contain many incorrect structures, mainly due to the alignment problems in the threading algorithms, resulting in incorrectly paired tertiary contacts or wrong secondary structure assignments. In contrast, Park & Levitt decoys' set [22] was generated by means of a lattice modeling with constrained native secondary structure and covered wide range of RMSD values. Decoys built by Rosetta from Lee et al. work [8] exhibited varying topologies with locally optimized structure. The size of this set (a small number of small proteins) is probably not sufficient for a clear estimation of the correlation between RMSD and energy. That makes such decoys' sets less challenging then those investigated in the current work. While threading methods are limited to the existence of structural analogs, high-resolution lattice models can be used efficiently in comparative modeling as well as in *de novo *structure predictions [18].

**Figure 1:**
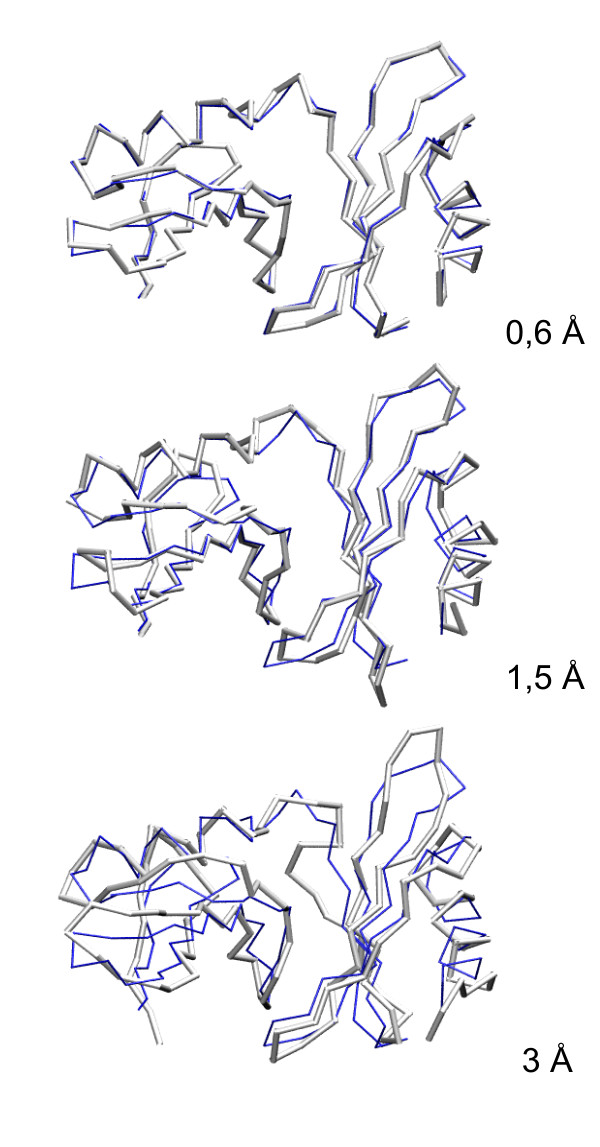
**Accuracy-representative models from the CABS decoys set**. Three 2GU3A example models with various distances from the native structure (the lowest energy model – 0.6 Å, intermediate 1.5 Å, and the worst one 3 Å from native). Models are plotted in gray, reference native structure in dark thin line.

**Figure 2:**
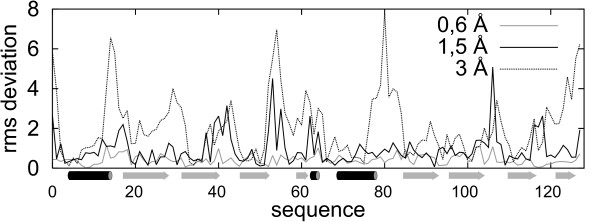
**Illustration of the secondary structure dependent character of differences between the accuracy-representative models**. RMSD deviation from native for each residue of three 2GU3A example decoys (after the best superimposition of the entire structures – see Figure 1). On the sequence axis the secondary structure is symbolically depicted (helices in black and strands in grey).

In this work, long Monte Carlo simulation with the CABS model [17] were performed in order to generate protein-like near-native decoys with RMSD in the range of 0.35 – 3 Å from native (0.35 Å is the average accuracy of the Cα-trace projections onto the CABS underlying lattice grid). The CABS model features high computational efficiency and has the ability to cover the near-native conformational space (given some constraints extracted from a correctly aligned template) or can be used in *de novo *structure prediction. As can be seen from the example given in Figure 1 and Figure 2, the near-native decoys generated by CABS consist of structures varying mainly in the most flexible regions as loops or near the ends of the secondary structure elements. We have decided to limit the range of the decoys diversity from about 0.35 to 3 Å from the native. This is a typical range for the comparative modeling.

The main objectives of the use of Molecular Mechanics force field after the coarse-grained stage modeling in our work are: improving filtering of the crude models, providing better correlation with similarity to the native, and then identification of the best model (closest to the native). To reliably verify the correlation between the Molecular Mechanics energy and RMSD we decided to divide each protein subset (60 – 150 thousands of structures, depending on a protein) onto 30 bins, using RMSD from the native structure as a criterion for the classification (from 0 to 3 Å, with the bin size of 0.1 Å). From each bin, 30 models or less if there weren't as many, were randomly selected. In this manner approximately 800 decoys were selected for each protein, with a broad spectrum of the quality of models (in the sense of similarity to the native structure).

### From simplified to all-atom representation

Employing simplified protein representation for exploration of the vast conformational space (including *de novo *structure prediction, various comparative modeling techniques, or hybrid methods utilizing different kinds restraints from experimental data) brings the necessity of reconstruction of the reduced models to the all-atom representation, compatible with the classical all-atom modeling tools [21]. Rebuilding procedure may also be beneficial when structures from different sources (and of different quality) are being compared [10]. Recently, during the extensive tests of available methods for reconstruction of protein backbone from Cα-trace [21], we found that in the cases of the high accuracy models (better than 1.5 Å) the best performance is achieved by the procedure employing insertion of well adjusted fragments from known protein structures [26] (implemented in the Sybyl software from Tripos Inc. St. Louis, MO). Such procedure improves the local geometry of the backbone. To assure the best possible reconstruction, we applied this method to our benchmark set. Similarly to the main-chain backbone atoms, the side-chains were reconstructed and their conformations optimized using Sybyl. It is worth noting, that the increase of the number of experimentally determined high-resolution structures in the Protein Data Bank (PDB) may lead to further improvements in the all-atom reconstruction methods that use protein fragments from the PDB.

### CABS decoys evaluation by all-atom minimizations

Evaluation of protein models were done by all-atom minimization with frozen alpha carbons using Amber7 FF99 force field and Amber charges [27] implemented in Sybyl. The effect of solvent has been neglected and a uniform value of the dielectric constant was set equal to 1. Due to the frozen positions of the alpha carbons, this is probably an acceptable approximation. What important, it is often unknown whether the target sequence is a part of a larger oligomeric structure [12]. If it is, the solvation energy term would unnecessarily penalize for the exposed binding part of the protein surface. Moreover, the ranking of large sets of decoys of relatively large molecules requires as fast as possible computations. That would be impossible, or very difficult, with the explicit treatment of the surrounding solvent [2]. Also, the fixed positions of the alpha carbons prevent from evolution of the all-atom systems into directions of non-native local minima. On the other hand, a significant repacking of the model structures is rather unlike within the frozen Cα approximation. The underlying assumption is that the set of decoys contains a fraction of a good-geometry near-native structures.

**Figure 3:**
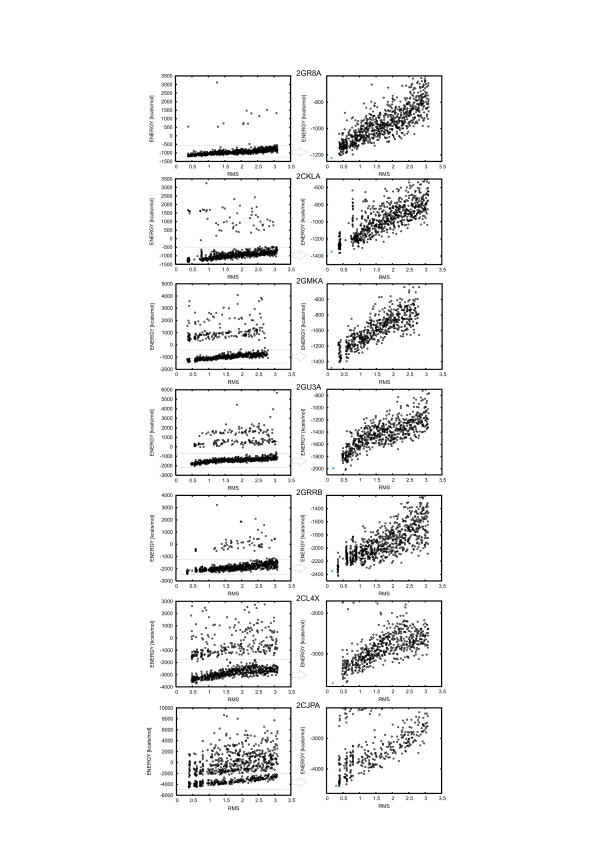
**Results of 1000 iteration minimization for the CABS decoys**. For each protein, the energy was plotted as a function of Cα RMSD for all decoys (left panels) and without decoys with abnormal high energy values resulted from structural inaccuracies (right panels). On the left panels, energies of the native structures are denoted by asterisks. The native structures were subjected to the same rebuilding procedure from the Cα-traces as that applied to the decoys. Proteins are ordered in respect to their chain lengths (Table 1) – from the smallest on top (2GR8A) to the largest (2CJPA) on the bottom.

The results of minimization are illustrated in Figure 3. For each protein, the decoys' energies after 1000 iterations of the Sybyl minimization were plotted as a function of the Cα-trace RMSD. For all proteins, resulting energies as a function of RMSD divide into two ensembles: wedge shaped low energy values (Figure 3, right panels) and abnormal high energy values (Figure 3, left panels). The abnormal high energy values, observed for a fraction of the decoys, resulted mainly from bond stretching and the van der Waals repulsive energy contribution due to the rebuilding inaccuracies leading to the overlaps of some atoms. The decoys were produced by the low resolution search, with a very simplified representation of the side chains. This flattens the energy landscape but it also may result in a distorted geometry of the Cα-trace. This is in the agreement with the observation that physic-based energy functions are sensitive to small displacements as opposed to the statistical energy functions [28]. The rebuilding procedure aimed at adjustment of protein fragments as closely as possible to the initial Cα trace, and consequently was not always capable of constructing structures without some local defects. Structures with small errors can be easy filtered out by a short minimization – range of 200 iterations, regardless of the protein length. This is sufficient to reject the decoys with the local defects (energy > 0) and it takes about 5 minutes per one structure of a large protein domain (2CJPA) on a single LINUX box. Interestingly, in all cases such short minimization leads practically to the same correlation between energy and RMSD as a 5 times longer minimization (e.g. the Pearson's correlation coefficient was equal to r = 0.79 for 2GR8A models that were scored in the negative energy range after 200 iterations, and for the same models r = 0.80 after 1000 iterations procedure). However, we found that while in the case of the high accuracy decoys a longer minimization didn't bring any substantial changes to the ranking, for filtering out the medium accuracy decoys (in the range from 2 Å to 3 Å) from the worse models, a longer minimization (1000 iterations) led to better results in the identifying the best model (see the section on Evaluation of the MOULDER testing set).

The number of steric mistakes grows with the protein length (Table 1, Figure 3). The exception is 2GRRB, an all-alpha protein which was rebuilt the most accurately from the whole set. While clashes could be easily removed via a short relaxation of the entire structures, constructing instead a larger number of the reduced space decoys (and rejecting these with clashes) seems to be a more effective option.

Figure 3 clearly shows that the proposed procedure leads to the proper ranking of models – there is very good correlation between energy and the RMSD from the native structure. The lowest energy models are always very close to the native and in most cases the best decoys have been selected.

At this point it should be added, that there is nothing specific about the decoys generated by CABS with the subsequent all-atom rebuilding. The CASP6 assessments have shown, that the local geometry of the CABS models was on average the same as the local geometry of the models built by means of other high-performance methods for protein structure prediction. This is mainly due to the fact, that various all-atom reconstruction procedures employ in a similar fashion protein fragments extracted from the high-resolution crystallographic structures. Thus, the proposed method should work similarly well for decoys generated by means of different modeling algorithms.

### Evaluation of the MOULDER testing set

To test the ability of our protocol to discriminate a medium-accuracy models (better than 3 Å), from a low-accuracy models we used MOULDER decoys' set, evaluated by Eramian et al. [12] using 24 individual assessment scores, including physic-based energy functions, statistical potentials, and machine-learning scoring functions. Each of the targets from the set was modeled using a template of <30% sequence identity, corresponding to challenging comparative modeling cases. No two alignments of a given target shared >95% of identically alignment positions. The target-template alignments were obtained using MOULDER [29] with MODELLER [20] to create 300 different target-template alignments.

Of the 20 targets subsets, only 7 contain models better than 3 Å (for RMSD range and median RMSD see Table 2). We decided to reduce the testing set to these 7 proteins, since the sensitivity to small structural displacement make physical force-fields less suitable for the assessment of models with larger errors [28]. Before the minimization procedure, coordinates of the alpha carbons of the models were extracted and subjected to rebuilding procedure, identical to the one applied to the CABS decoys' set.

**Table 2:** The 7 protein subsets from the MOULDER testing set

PDB ID	SS type	L	RMSD range	Median RMSD	*avg*.Δ*RMSD (best, rank.)*	Δ*RMSD*
2mtaC	α	81	2.2–42.7	6.7	0.56 (0.30, **5**)	0.26
1onc_	α, β	101	2.2–22.8	10.5	0.37 (0.25, **8**)	0.30
1bbhA	α	127	2.5–20.8	6.5	0.51 (0.05, **17**)	0.76
1mdc_	β	130	1.9–16.4	9.3	0.37 (0.13, **8**)	0.02
1dxtB	α	143	2.0–34.1	7.2	0.56 (0.31, **3**)	0.08
2fbjL	β	210	2.4–22.5	8.8	1.51 (0.32, **19**)	0.53
2cmd_	α, β	310	2.5–20.2	5.8	1.26 (0.31, **13**)	1.58

Particular columns contain: the PDB code, the secondary structure type, the protein length, the range of Cα RMSD (Å), the median of RMSD (Å), the average ΔRMSD of our method, in the brackets: the average ΔRMSD of the best method [12] and a ranking – the number of methods that outperformed our procedure (23 individual assessment methods were tested, SVMod that uses a composite score from the individual methods was not taken into account [12]), the ΔRMSD on the whole subset.

The performance of the methods with the MOULDER decoys' set were measured by average RMSD difference (ΔRMSD) between the model identified as the best of the set and the model with the lowest RMSD. Each of the sets of 300 models was split into 2000 randomly populated smaller sets of 75 models. The purpose of this division was to reduce the impact of individual target sets on the final ranking and to increase the robustness of the benchmark. For each 75 model set, the model with the lowest Cα RMSD (after superposition with the native structure) was used as a reference to calculate the ΔRMSD measure. We followed the same rules, despite the fact that the average ΔRMSD value of the 75 model set is not suited for evaluating of our procedure, which is aimed at assessing much larger sets due to its characteristics. Our method narrows down the number of models in the testing set rejecting the fraction of them (extreme high energy values), due to their small inaccuracies. Sensitivity for the small displacements is the price for the high discriminatory power [28]. Additionally, due to the same reasons, it is desirable to provide a few copies of the model with the small differences, to maximize the chance of the accurate scoring. Such sets of models (clusters), representative for a various type of conformations, can be easily extracted from reduced modeling trajectories by structural clustering [30] and subjected to the evaluation procedure.

Two worst cases, which are the two largest proteins from the set, illustrate the effect of narrowing down the number of models and insufficient number of good models in the set. The former situation is observed for the subset of 2fjbL models, where only one third had been the subject to the 1000 iteration minimization, while the rest was rejected after 200 step minimization (E>0). The latter could be observed in the case of the 2cmd subset of models, which is the subset with the smallest number of models better than 3 Å (16 out of 300).

Omitting these two worst cases, our method performed similarly to the Rosetta scoring function which is apparently the most successful in *de novo *high-resolution small protein structure prediction [2]. ΔRMSD value averaged for the 5 subsets of proteins was 0.47 for our procedure (for individual subsets values see Table 2) and 0.49 for the Rosetta. Corresponding values for two physic-based approaches used in the study by Eramian et al. were 0.56 and 0.51 for GB (CHARMM with Generalized Born solvent model) and EEF1 (CHARMM EEF1), respectively [12]. The authors noted that in the selecting the best model from a set of very similar models EEF1 and GB were more accurate than many of the statistical potentials tested. According to their suggestion it is possible that different relaxation schemes would have produced better results. They took also into consideration, that by including the solvent model, the oligomeric proteins were presumably harder to evaluate than monomers.

The ability of the all tested methods to identify native-like models greatly varied across different targets [12]. The most accurate methods that obtained the best results for the 7 targets considered here (see Table 2 for the best avg. ΔRMSD) were: DFIRE, MODCHECK and MODPIPE_COMBI implementing different kinds of statistical potentials and PSIPRED/DSSP (score based on predicted secondary structure using PSIPRED, compared with the model secondary structure assigned by DSSP algorithm) [12].

**Figure 4:**
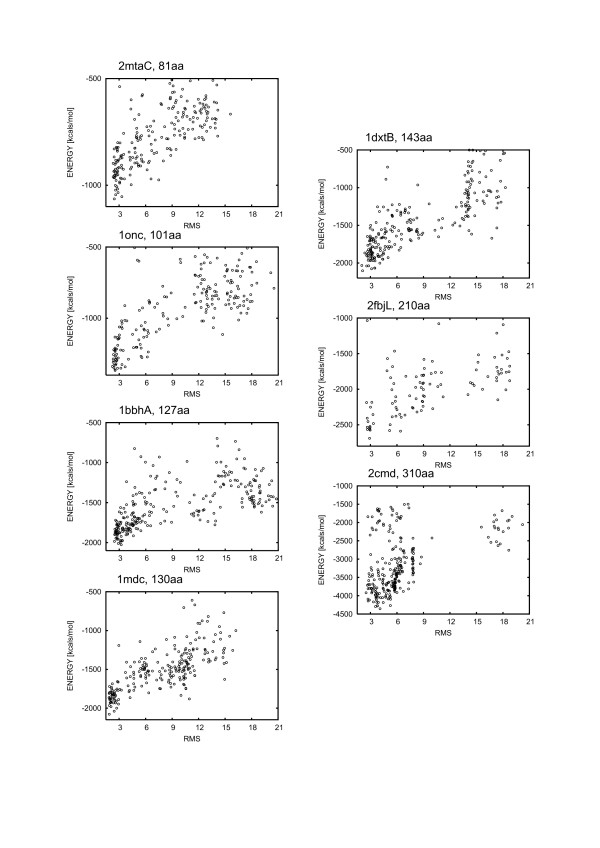
**Results of 1000 iteration minimization for the Moulder decoys**. For each subset of decoys, the energy was plotted as a function of Cα RMSD for the best scored decoys.

As we said earlier, the minimization with frozen Cα has to be performed on a sufficient number of models. The number of models in the studied MOULDER subsets (300) seems to be enough (Table 2 and Figure 4). Obtained ΔRMSD on the whole subsets surprisingly well correlate with the minimum values of the RMSD (Table 2), confirming the usefulness of our procedure in the high-accuracy modeling protocol. Clearly, performance of our methods improves with increasing average quality of the decoys. Thus, the analysis of the MOULDER decoys indicates that the best results of the proposed procedure are expected for the sets of relatively good models. This is actually a nice finding, since ranking of very bad models isn't useful anyway. It is also worth to mention that the final selection can be likely improved by a structural clustering of the best scored models.

## Conclusion

In summary, the proposed simulation protocol makes possible fast and reliable assessment of high resolution structures for relatively large proteins. Bradley et al. [2] have shown recently that a high resolution *de novo *structure prediction can be achieved for small proteins by using an all-atom refinement procedure in the last stage of prediction. The cost of application of the high resolution refinement for large proteins was estimated by authors to require orders of magnitude more computing power, than the 150 CPU days required for small proteins [2]. A single, 500-step, minimization sufficient for the ranking of the smallest proteins (of a comparable size to those from Bradley et al. work) took in present work approximately 1.5 minutes. The approach described here may be a good alternative for the refinement of small proteins and could be applied as a means of the best model selection in a large scale modeling, regardless of protein length.

Protein model filtering in the endgame of protein structure prediction protocols faces the following two challenges: fold identification (particularly in *de novo *modeling) and the selection of the best models from a set of good models (especially important in comparative modeling). The results of this work apply mostly to the model selection in comparative modeling. Recent CASPs test have shown that the best comparative models are built with a lot of human intervention using an assortment of well known modeling tools [1]. The challenge is to automate the protein prediction and produce even more accurate models with no need of the human assistance during the prediction protocol. Thus, consistent and accurate last stage of modeling is needed, i.e., producing and filtering the high-resolution predictions. Elaborate human intervention can be compensated by a high-throughput modeling, employing sampling of alternative alignments (which should lead to a sufficient sampling of the near-native regions of the conformational space) and an efficient scoring of the large number of obtained decoys.

The need for a reliable detection of the best native-like models from a set of different predictions produced in the recent CAFASP4 experiment (CAFASP assesses the performance of methods without the user intervention allowed in CASP) led to a new category of model assessments – Model Quality Assessment Programs (MQAPs). Although performance of currently available MQAPs indicate that some of these methods may be useful for new automated procedures, high false positive rates are observed, and the MQAP methods were suggested to be used as additional elements of the prediction protocols rather than as a simple post-filter [31].

Our method gives surprisingly consistent correlation of the all-atom energies with RMSD distance from the native structure. Decoys within 3 Å from native were examined and no false positive cases were noted. As mentioned before, the correlation of the CABS energy with RMSD in this range is often insignificant. The starting energy of the all-atom structures are uncorrelated with the CABS energy and are extremely high, mostly due to the overlaps of the side chains. The Molecular Mechanics energy decreases rapidly during the initial stage of minimization, mainly due to the improvement of side-chain rotamers position. For a fraction of decoys (see Table 1) the energy plateaus at a high level due to non-resolvable steric clashes. The subset of the low-energy structures, easily distinguishable from the high-energy ones, exhibits the above mentioned, nearly perfect correlation with the values of RMSD from the native structure. This provides a very strong support for the idea of multiscale high-resolution protein modeling. More extensive molecular dynamics simulation, than described here, might lead to even better model ranking and refinement. In the case of fixed Cα-traces, a longer than performing 1000 iterations minimization is not necessary – the results do not change anymore.

Finally, we would like to return to the two important assumptions of the present method: fixed positions of the alpha carbons during the minimization and the in vacuum Molecular Mechanics. Obviously, these assumptions significantly reduce the cost of computations for large sets of decoys. The frozen alpha carbon approximation works very well for the model ranking, although it eliminates possibility of a significant refinement of the entire structures. Model ranking exercises performed on the MOULDER set by others have shown clearly that there is very little added value with use of more rigorous Molecular Mechanics procedures [12]. The same conclusion could be drawn from our experiments with defrosted alpha carbons and with a continuous model of solvent (unpublished). The ability to generate sets of decoys containing a significant fraction of the near-native structures by coarse-grained modeling (followed by the all-atom refinement and model ranking) remains a key factor for the high-accuracy structure prediction.

## Methods

The proposed procedure is a combination of tools available in the BioShell package [32] and commercial Sybyl software (Tripos Inc. St. Louis, MO) integrated in a single pipeline with the reduced-space CABS [17] protein folding algorithm, which can be employed in a high-throughput protein modeling.

### Decoys generation by CABS

The CABS model uses a lattice representation with 800 possible orientations of the virtual alpha carbon-alpha carbon bonds [17]. The sampling scheme of the conformational space employs the Replica Exchange Monte Carlo method. Knowledge-based potentials of the force field include: generic protein-like conformational biases, statistical potentials for the short-range conformational propensities, a model of the main chain hydrogen bonds and context-dependent statistical potentials describing the side group interactions. The model could be effectively used for high resolution comparative modeling as well as for purely *de novo *folding of small globular proteins.

The Bioshell package was very useful in managing and analyzing large volume of simulation data.

### Backbone reconstruction

Reconstruction procedure from alpha carbons to backbone, implemented in Sybyl/Biopolymer, with default settings was used. Procedure bases on a "spare parts" approach [26], using fragments retrieved from the protein database (PRODAT) to construct the full poly-alanine backbone. Subsequently side chains were added by the standard procedure implemented in Sybyl/Biopolymer with the initial side chain position from the Sybyl database.

### All-atom minimization

We run all-atom minimization for all decoys (over 5500 structures from the CABS decoys' set and 2100 from the Moulder testing set) with frozen alpha carbons using implemented in Sybyl Amber7 ff99 force field, Amber charges, dielectric constant equal 1.0 and Powell minimization method, without initial optimization.

Amber7 ff99 energy expression:

E_total _= E_str_+ E_bend _+ E_tor _+ E_vdw/ele_, (E_str _- Bond Stretching Energy Term, E_bend _- Angle Bending Energy Term, E_tors _- Torsional Energy Term, E_vdw/ele _- van der Waals/Electrostatic Energy Term)

Maximum number of iterations was set to 5000, however the simulations longer than 1000 iterations were not necessary. To speed up maximally the calculations, a sufficient number of iterations (resulting in model ranking similar to the unlimited minimization) should be range of 500–1000, or even shorter, depending on the accuracy of models.

CABS and Bioshell package can be downloaded from our website [33].

## Authors' contributions

SK performed the backbone rebuilding procedures, Molecular Mechanics, statistical analysis, assembled figures and wrote a draft of the manuscript. DG constructed the CABS decoys' set. AK conceived the study, coordinated it and wrote the final manuscript. All authors participated in the design of the study, read and approved the final manuscript.
